# Integrating mammography screening programmes into specialist breast centres in Italy: insights from a national survey of Senonetwork breast centres

**DOI:** 10.1186/s12913-022-08111-1

**Published:** 2022-05-31

**Authors:** Silvia Deandrea, Francesca Ferrè, Rosanna D’Antona, Catia Angiolini, Marina Bortul, Lauro Bucchi, Francesca Caumo, Lucio Fortunato, Livia Giordano, Monica Giordano, Paola Mantellini, Irene Martelli, Giuseppe Melucci, Carlo Naldoni, Eugenio Paci, Loredana Pau, Gianni Saguatti, Elisabetta Sestini, Corrado Tinterri, Milena Vainieri, Luigi Cataliotti

**Affiliations:** 1 Environmental Health Unit, Agency for Health Protection, Pavia, Italy; 2 Department EMbeDS, Management and Health Laboratory, Institute of Management, Sant’Anna School of Advanced Studies, Pisa, Italy; 3 Europa Donna Italia, Milan, Italy; 4 SOD Oncologia Della Mammella, Breast Unit, DAI Oncologico, Azienda Ospedaliero-Universitaria Careggi, Florence, Italy; 5 Breast Unit, Division of General Surgery, Azienda Sanitaria Universitaria Giuliano Isontina, Hospital of Cattinara, Trieste, Italy; 6 Romagna Cancer Registry, Romagna Cancer Institute, IRCCS Istituto Romagnolo Per Lo Studio Dei Tumori (IRST) Dino Amadori, Meldola, Italy; 7 Department of Breast Radiology, Veneto Institute of Oncology, IRCCS, Padua, Italy; 8 Breast Centre, San Giovanni-Addolorata Hospital, Rome, Italy; 9 CPO Piedmont, AOU Cittá Della Salute E Della Scienza, Turin, Italy; 10 Medical Oncology Department, Azienda Socio Sanitaria Territoriale Lariana, Como, Italy; 11 Screening Unit, ISPRO - Oncological Network, Prevention and Research Institute, Florence, Italy; 12 SS Radiologia Senologica, ASL ‘SS. Annunziata’, Taranto, Italy; 13 Italian Group for Mammography Screening, Florence, Italy; 14 Senology Unit, Local Health Authority, Bologna, Italy; 15 Breast Unit, Humanitas Cancer Centre, Rozzano, Milano Italy; 16 A.P.S. Senonetwork Italia, Florence, Italy

**Keywords:** Mammography screening, Breast centre, Health services integration, UTAUT, Survey

## Abstract

**Background:**

Despite recommendations, mammography screening is often insufficiently integrated into specialist breast centres. A national, cross-sectional, voluntary, online survey on this issue was carried out among the Italian breast centres associated with Senonetwork, the Italian network of breast cancer services.

**Methods:**

A 73-item questionnaire was created, pre-tested and piloted. Centres integrating and not integrating a screening programme were compared using the unified theory of acceptance and use of technology (UTAUT) model. Centres’ clustering was performed using the Gower’s distance metric. Groups and clusters were compared with the equality-of-means test.

**Results:**

The response rate was 82/128 (65%). Overall, 84% (69/82) breast centres reported a collaboration with a screening programme in performing and/or reading mammograms and in the diagnostic work-up of women with abnormal screening results. The same proportion was observed among those centres responding to all questions (62/74). Performance expectancies (or the perceived usefulness of integration in terms of clinical quality, patient convenience, ease of job, and professional growth), satisfaction and motivation were higher in those centres collaborating with the screening programme. Effort expectancy indicators (or the degree to which the respondents believe that the integration is easy to implement) and those concerning the existence of facilitating conditions were lower both in centres collaborating and not collaborating with the screening programme. Among the former, six clusters of centres, distributed from ‘no integration’ to ‘high’, were identified. In cluster analysis, the highest level of integration was associated with higher agreement that integration eases the job, offers better opportunities for professional growth, and makes the working environment more satisfactory. The least integrated cluster assigned the lowest score to the statement that local health authority made available the resources needed.

**Conclusions:**

While confirming the positive effects of integrating screening programmes into breast centres, this survey has brought to light specific difficulties that must be faced. The results provide insights into the importance of integration focusing on the perspectives of professional career and motivation. The deficiency of facilitating conditions to integration is modifiable. Screening professionals’ societies may have a role as initiators of the integration. Other supporting actions may be included in health laws at the national and regional level.

## Background

### State of the relationship between breast centres and screening programmes in Europe

A breast centre (also referred to as breast unit) is defined as the place that provides all breast care services on a multidisciplinary basis, including genetics and prevention, treatment of primary tumour, care of advanced disease, supportive and palliative care, survivorship care and psychosocial support [1]. Since over 20 years, there is evidence that patients treated and cared for by specialist teams in dedicated breast centres have better outcomes [2].

The EUSOMA (European Society of Breast Cancer Specialists) position paper in which the correct standards for the set-up of breast centres were first issued was published in 2000 [3]. In 2003 and 2006, two resolutions on breast cancer of the European Parliament recognised the EUSOMA requirements for high-quality breast centres and set the deadline of 2016 for their creation [4, 5]. The deadline, in fact, has been missed by most Member States of the Union [6]. A survey by the Joint Research Centre of the European Commission has shown that there remains a great deal of diversity in the implementation of the EUSOMA requirements [7]. The main factors accounting for this include differences in type of healthcare system, presence, rationale and implementation of national and regional cancer plans, amount of financial resources, accreditation rules, level of multidisciplinary expertise and rate of average adherence to guidelines. As a result, there is still a wide variation in breast cancer diagnosis, treatment and outcomes both between and within the European countries [6].

This is also the case for the integration of mammography screening programmes into breast centres [8]. The EUSOMA recommends that organised screening activities should be based within, or very close to, breast centres [9]. The justifications include the convenience and comfort of women, the role played by radiologists in both screening and diagnostic imaging, and the facilitation of communication between all professional involved in the diagnostic work-up of women with screen-detected breast abnormalities [1, 6, 9].

Screening providers have suggested other –and equally compelling– reasons for recommending that mammography screening services be part of breast centres [10]. First, the screening process is inherently multidisciplinary and the multidisciplinary work experience acquired with organised screening programmes would make a valuable contribution to the creation of breast centres. Second, breast centres are responsible for breast cancer control on a population basis and, currently, organised screening programmes are the only formally population-based breast care services in Europe. Screening providers might bring into breast centres the idea that only a high degree of attendance, i.e., of women’s access and population coverage, enables breast care services to make an impact on death rates. And third, the EUSOMA requirements for breast centres include systematic collection and analysis of service data [1, 9]. Mammography screening programmes are the object of an intensive monitoring work, and their information infrastructure may provide a useful framework for processing, transmission, and storage of vast amounts of clinical data.

Despite this, another Joint Research Centre survey has reported that, in 40% of the EU countries, the processes and services constituting the breast cancer care pathway differ in the level of supervision for organisation and quality by an external authority [11]. The level varies especially for screening programmes, because these are usually organised separately.

In Italy, the situation is ill-defined. Although the Italian Group for Mammography Screening (GISMa, the scientific society representing the screening programmes) endorses the recommendation of the EUSOMA [10], there are only anecdotal data about the integration of mammography screening programmes into breast centres [12]. For this reason, the GISMa, Senonetwork (the Italian network of breast cancer services), and Europa Donna Italia (the Italian section of the Europa Donna, the advocacy organisation for breast cancer patients in Europe) undertook a national, cross-sectional, voluntary, online survey among the Italian breast centres, with the general objectives to cluster them according to degree of integration with mammography screening programmes and to explore the perception of utility, effort required, acceptability of the integration, and facilitating conditions among the professionals involved.

### The conceptual framework of the survey

The survey was designed considering that (i) the process of creation of breast centres, in Italy and elsewhere, is incomplete [6] and qualitatively heterogeneous [7], (ii) the level and type of integration of the screening programmes adds further diversification, and (iii) a thorough analysis is needed to deepen the understanding of the related problems and –more important– of the related expectations and opportunities for fostering such integration. The latter two are pivotal elements in the process of facilitating the organisational innovation and the acceptance and use of new models.

Integration of care is a multidimensional concept that has received enormous attention in the health care research domain. More than 170 overlapping definitions are available [13] making it hard to conceptualise. The World Health Organisation (WHO) defines integrated care as “ … a system that brings together inputs, delivery management and organisation of services related to diagnosis, treatment, care, rehabilitation and health promotion […] to improve access, quality, user satisfaction and efficiency” [14]. Evidence suggests that transformations towards integrated care require a good understanding of the various dimensions of integration, which in effect calls for the development of a comprehensive overview. Drawing from the definition provided above, taxonomies of integrated care can be distinguished between organisational, functional, service, and clinical [15, 16]. Organisational integration can be referred to as bringing together units, service departments or organisations through coordinated actions of shared planning and regulation to provide joined-up, personalized care. Types of integration include networks and mergers. Functional or administrative integration means integration of non-clinical and back-office or support functions through, for example, shared electronic patient records, common clinical databases or information systems. Service integration refers to integration of different clinical services at an organisational level by, for example, establishing multidisciplinary or cross-functional teams. Clinical integration is integration of care into a single and coherent process within/or across professions by means of, among others, using shared guidelines and protocols such as diagnostic, therapeutic and care pathways. Based on this taxonomy, a section of the survey was developed to explore the type of integration established between screening programmes and breast centres.

Moreover, the survey included items aiming at investigating the perception of professionals in working through the integration process. Perception (human factors) has considerable relevance in the processes of acceptance and facilitation of the introduction of changes in work practice because expectations have a significant impact on behaviour. Exploring the mechanisms that influence the intentions to implement specific behaviours and the behaviour itself is useful for bringing out what can be the determinants of a high/low level of acceptance of change. In the literature, the model of the Unified Theory of Acceptance and Use of Technology (UTAUT), developed through the revision and integration of eight dominant theories and models of the behavioural and organisational sciences, offers an interesting reading on the intentions to implement specific behaviours (behavioural intention to use, BI) and the behaviour itself (use behaviour) [17]. According to the UTAUT scheme, there are four main predictors of user behaviour, i.e., the BI and the use behaviour are determined by four main constructs: performance expectancy, effort expectancy, social influence and facilitating conditions. The influence of these key factors on the intention of the behaviour and the behaviour itself is moderated from time to time by other characteristics such as gender, age, experience and voluntary use. It is believed that by examining the presence of each of these constructs in the “real world” it is possible to assess the intention to use a specific system at a single individual level.

The performance expectancy represents how much an individual believes that technology will help him to obtain advantages in carrying out his work, also in terms of greater usefulness and better performance results. The effort expectancy is understood as the level of ease of use of the technology and represents how much an individual believes that the use of technology is easy. Social influence is defined as the degree to which an individual believes that those who are important in the context of implementing the technology believe that the technology should be used and strongly recommend it. In fact, it represents the positive influence that those deemed influential have with respect to the use of technology. The facilitating conditions are defined as the degree to which an individual believes that the technical and organisational infrastructure exists to support the use of the technology. In the UTAUT model, the first three constructs, namely performance expectancy, effort expectancy, and social influence, have an effect on the intention to use the technology, while actual use is conditioned by the intention to use and the facilitating conditions.

The UTAUT model has been developed and widely used to analyse the level of acceptance and use of innovations in different fields and sectors [18], including the health sector [19–22]. This framework can be used to evaluate user expectations with reference to specific innovations, including organisational ones, because it incorporates theories of human behaviour and allows analysing the perceptions of subjects with reference to a broader concept of innovation. In the survey reported here, two items about how respondents evaluate the experience of integration were added in order to explore the relationship with the constructs of the UTAUT model. In addition to the verification of the UTAUT model for the analysis of the mechanisms of acceptance of the change under observation, the study hypothesis was –in fact– to verify whether performance expectancy, effort expectancy, the social influence and the facilitating conditions are associated with the satisfaction of integration among professionals.

## Methods

### Objectives

The quali-quantitative analysis reported in this article was specifically aimed (i) to determine the proportion of breast centres collaborating with a screening programme, and to evaluate the correlates of collaboration versus non-collaboration, (ii) to cluster the breast centres collaborating with the screening programme based on the taxonomy of integration, and (iii) to identify the correlates of these clusters.

### Setting

In Italy, the national legal framework implementing the 2003 and 2006 resolutions of the European Parliament [4, 5] was issued in 2014. Healthcare services, however, are governed by the regional administrations, which are responsible both for the creation of breast centres –or appointment of existing centres– and the development and application of the related criteria. Consequently, these vary across the country, and this also holds true for the modes of integration –if any– of screening programmes. It must be noted that, in Italy, organised mammography screening is included in the essential package of health services that should be provided to the population by the regional health systems. The degree of implementation of this plan is centrally monitored [23].

### Survey development

The project was developed and implemented following the survey conduction best practices proposed by Burns et al. [24]. The questionnaire was created eliciting appropriate themes from the national stakeholders. Account was taken of (i) the relevant national legislation, (ii) the international literature pertaining to the requirements of breast centres [1, 6, 7, 9] and to the taxonomy of integration of health services [15, 16], and (iii) a modified version of the UTAUT framework [17].

The questions on the integration of screening programmes into breast centres were developed using an adaptation of the taxonomy proposed internationally [15, 16]. A modified version of the UTAUT model was used that included six constructs, namely: performance expectancy, effort expectancy, social influence, facilitating conditions, propensity to use, and satisfaction/motivation. Through these key concepts, the UTAUT explains the intentions to use any given technology (in our case the integration and the related opportunities) as well as the resulting usage behaviour [17].

The final version of the questionnaire consisted of 73 questions under the following domains: (i) breast centre’s identification and main characteristics including, among others, the certification according to the EUSOMA standards [25] (questions 1–16); (ii) breast centre clinical lead’s perception of utility, effort required, acceptability, and facilitating conditions of the integration of the screening programme into the breast centre (17–34); (iii) breast centre’s screening mammogram volume and relationship with the screening programme (35–48); (iv) dimension of integration: organisational (49–51), functional (52–58), service-related (59–64), and clinical (65); and (v) structural and functional details of the integration (66-73). All respondents were meant to go through questions 1–16 and then to a skip logic question asking whether the breast centre collaborates with the screening programme. In case the answer was no, the breast centre was asked an additional multiple-choice question about the reasons for that, then it went to questions 17–34 and finished the survey. In case the answer was yes, the breast centre responded to questions 35–73 about the characteristics of such integration. Eventually, questions 17–34 were proposed at the very end of the survey.

The questions on expectancies, social influence, facilitating conditions, propensity to use, and satisfaction/motivation were treated as statements. Respondents were asked to express their level of agreement with them on a 0–100 scale.

A PDF version of the questionnaire (in Italian) is available at the website of the GISMa [26].

An ancillary article reporting on a secondary endpoint, that is, the provision of follow-up care for women with a history of breast cancer, has been previously published [27].

### Survey pre-testing and piloting

Before full deployment, the survey was pre-tested on a sample of three breast centres from different administrative regions – supposedly characterized by different breast centre models. Based on their feedback, we refined and clarified unclearly-worded, ambiguous, misleading, or non-relevant questions. The survey was subsequently loaded onto the SurveyMonkey platform (https://it.surveymonkey.com/) and successfully piloted by one volunteer breast centre for technical functionality of the online instrument.

### Eligibility criteria

At the time the survey was designed, the creation of breast centres was still incomplete in some administrative regions. As a consequence, the official national list of breast centres –the potential targets of the survey– had not been published yet. This problem was dealt with by (i) targeting the centres associated with Senonetwork, which number is known, in order to calculate a response rate and (ii) using the proportion of participating centres that reported being appointed by a regional administration as an indicator of unselected composition of respondents.

### Survey process

An invitation to participate was sent via e-mail to the clinical leads of eligible breast centres, or the main contact persons. The e-mail contained a link to the online instrument. The survey was conducted between July 2020 and October 2020. To increase the response rate, a reminder e-mail was sent. No financial incentives were offered.

### Data analysis

For the purposes of the present analysis, selected items from the questionnaire were used. Standard statistics, including frequencies, proportions, medians, ranges, and interquartile ranges, were used to summarise the characteristics of respondents to the survey.

For the items developed under the UTAUT framework on the perception of utility, effort required, acceptability of the integration, and facilitating conditions, the homogeneity of the mean of the reported scores from 0 to 100 between breast centres integrating and non-integrating a screening programme was evaluated with the equality-of-means test. To rule out confounding, the two groups were compared for a set of control variables including the number of years of professional experience of the clinical lead, the number of working years at the breast centre, age, and gender.

Clustering of respondents was performed using the Gower’s distance metric. The choice of the number of clusters was made by using the agglomerative clustering, which minimises the distance between elements of each cluster, and by visually inspecting the results on the dendrogram. The variables included to classify the clusters were based on the dimensions of integration [15, 16], that is, the functional integration, the clinical integration, and the structural integration. Table 1 shows the variables used for each characteristic. The equality-of-means test was performed to compare the answers between the clusters identified.

**Table 1:** Variables used in the cluster analysis by type of integration of screening programmes into breast centres

Type of integration	Variable
Structural integration	The breast centre and the screening programme use the same software for patient management (screening invitation, basic test, assessment examinations) (D52) The breast centre and the screening programme share a database with patient information (D55) There is a reference person who ensures the link between the breast centre and the screening programme for women with suspected cancer (D57) Number of activities shared by the breast centre and the screening programme using the same software for patient management (D53_CONT) Number of items of information shared between the breast centre and the screening programme via shared database (D56_CONT) Number of health workers/professionals who ensure the link between the breast centre and the screening programme (D58_CONT) Number of health professionals from the screening programme who also work in the clinical area of the breast centre (D59_CONT)
Process integration	The screening activity is included into the diagnostic-therapeutic clinical protocol adopted by the breast centre (D65)
Functional integration	The breast centre and the screening programme share the same budget (D51) The person responsible for the breast centre and the one responsible for the screening programme share objectives about responsiveness and promptness of treatment (D67) Frequency of coordination meetings between the breast centre and the screening programme (D70) Availability of training opportunities targeting health professionals both from the breast centre and the screening programme (D73)

For all analyses, the level of statistical significance was set at *P* < 0.10.

## Results

### Characteristics of responding breast centres

Of the 128 breast centres associated with Senonetwork on 1 July 2020, 82 (65%) replied to the survey and 74 (58%) responded to all questions. Fifty-three (65%) of total 82 questionnaires were from northern Italy. Respondents reported a median of 345 new breast cancer cases seen per year (interquartile range, 250–484). All of them but one (99%) reported a number of breast cancer cases seen per year > 150. The median number of breast radiologists was five (interquartile range, 3–7). The median number of mammograms per year was 15,000 (interquartile range, 9000–24,750). Sixty-one (74%) of respondents were regionally-appointed. There were 24 (29%) breast centres certified according to the EUSOMA standards.

### Proportion and correlates of breast centres collaborating with a screening programme

Overall, 84% (69/82) breast centres reported a collaboration with a screening programme – the same proportion observed among those centres responding to all questions (62/74). In Table 2, the characteristics of the 69 centres reporting a collaboration are compared with the characteristics of the other 13 centres. A collaboration was more common for centres treating an annual number of breast cancers below the median. Expectedly, the annual mammogram reading volume had an opposite effect. A positive association was also found for the BCCert certification. The questionnaire included a direct question on the reason for the absence of collaboration. The most common answer was that the decision was taken by the local screening providers (data not shown).

**Table 2:** Characteristics of breast centres collaborating and not collaborating with the screening programme (*n* = 82)

Characteristic	Total number	Collaboration with the screening programme		*P* value^a^
		**No (%)**	**Yes (%)**	
Geographic area				0.26
North	53	6 (11)	47 (89)	
Centre	19	4 (21)	15 (79)	
South	10	3 (30)	7 (70)	
Hospital classification				0.12
Public hospital	52	5 (10)	47 (90)	
Private accredited hospital	5	1 (20)	4 (80)	
IRCCS and AOU	14	5 (36)	9 (64)	
Private accredited IRCCS	11	2 (18)	9 (82)	
New breast cancer cases treated in the last year (n)^**b**^				0.067
< 345	41	3 (7)	38 (93)	
≥ 345	41	10 (24)	31 (76)	
Staff of the multidisciplinary team (n)^**b**^				1.00
< 21	41	7 (17)	34 (83)	
≥ 21	41	6 (15)	35 (85)	
Dedicated breast radiologists (n)^**bc**^				1.00
< 5	41	7 (17)	34 (83)	
≥ 5	41	6 (15)	35 (85)	
Dedicated radiographers (n)^**bd**^				0.23
< 5	43	9 (21)	34 (79)	
≥ 5	39	4 (10)	35 (90)	
Mammogram reading volume in the last year (n)^**b**^				0.068
< 15,000	42	10 (24)	32 (76)	
≥ 15,000	40	3 (8)	37 (93)	
Availability of a data manager				0.84
No	17	3 (18)	14 (82)	
Yes, external	4	1 (25)	3 (75)	
Yes, internal	61	9 (15)	52 (85)	
Availability of a clinical database for quality assurance and research				1.00
No	9	1 (11)	8 (89)	
Yes	73	12 (16)	61 (84)	
BCCert Certification				0.095
No	58	12 (21)	46 (79)	
Yes	24	1 (4)	23 (96)	

*BCCert* European Society of Breast Cancer Specialists’ Breast Centres Certification, *FU* follow-up, *IRCCS* Istituto di Ricovero e Cura a Carattere Scientifico (non-University Research Hospital), *AOU* Azienda Ospedaliero-Universitaria (University Hospital)

Some percentages add to more than 100% due to rounding

^**a**^For the chi-square test or (when appropriate) the Fisher exact test

^**b**^Dichotomised by the median value

^**c**^Defined as dedicating > 50% of working time to breast imaging and breast care

^**d**^Defined as dedicating > 50% of working time to breast imaging

### Analysis of expectations and conditions for integration

Seventy-four valid responses were analysed for these endpoints, including the 62 respondents declaring to have an integration with the screening programme and the 12 professionals who reported no integration. As shown in Table 3, the four performance expectancy indicators were largely and significantly higher in breast centres collaborating with the screening programme. Their clinical leads rated their agreement with the first three statements (the integration makes me more confident of the clinical quality of patient care and of patient convenience and eases my job) between approximately 83% and 96% versus 57% to 65%. Their agreement was particularly high with the statement concerning the clinical quality of patient care.

**Table 3:** Observed correlates and effects of, or expectancies from, the integration of screening programmes into breast centres according to clinical leads (*n* = 74)

UTAUT model construct Observed correlates and effects (or expectancies)	Mean (standard error)^a^		*P* value^b^
	**Breast centres collaborating with screening (*n* = 62)**	**Breast centres not collaborating with screening (*n* = 12)**	
Performance expectancy			
The integration makes (or I expect it makes) me more confident of the clinical quality of patient care	93.4 (1.80)	57.6 (11.58)	0.000
The integration makes (or I expect it makes) me more confident of patient convenience (service timeliness, etc.)	95.7 (1.59)	65.5 (10.68)	0.000
The integration eases (or I expect it eases) my job	83.0 (3.25)	65.1 (9.61)	0.039
The integration offers (or I expect it offers) better opportunities for my professional growth	73.7 (3.73)	56.6 (10.40)	0.079
Effort expectancy			
It is easy (or I expect it is easy) to acquire the management skills needed for the integration	55.3 (3.50)	53.8 (9.86)	0.87
Managing the integration does not cost (or I expect it does not cost) me extra working time	42.7 (3.86)	36.3 (6.59)	0.50
Social influence			
Do your colleagues think that the integration is important?	85.7 (2.61)	71.3 (11.41)	0.065
Does local health authority think that the integration is important?	76.2 (3.55)	66.0 (10.17)	0.27
Facilitating conditions			
Has local health authority made (or will it make) available to you the resources needed for the integration?	42.5 (4.38)	51.9 (10.91)	0.40
Has local health authority enabled (or will it enable) you to acquire the management skills needed for the integration?	51.5 (4.37)	47.8 (10.21)	0.74
Has local health authority developed (or will it develop) an official protocol for the management of breast cancer?	71.3 (4.25)	62.7 (11.58)	0.43
Propensity to use			
Are you inclined to handling the integration personally?	92.7 (2.16)	82.5 (8.53)	0.10
Are you inclined to keep on handle the integration personally and with conviction?	92.6 (16.52)	NA	
Satisfaction and motivation			
The integration makes (or I expect it makes) my working environment more stimulating	85.1 (2.54)	66.8 (10.56)	0.015
The integration makes (or I expect it makes) my working environment more satisfactory	84.2 (2.55)	60.5 (10.42)	0.002
*Control variables*			
No. of years of professional experience	28.4 (1.01)	27.9 (2.03)	0.84
No. of working years at the breast centre	15.7 (1.11)	13.3 (2.53)	0.39
Age (years)	58.1 (0.92)	55.5 (1.94)	0.24
Gender (female)	0.52 (0.06)	0.42 (0.15)	0.51

*UTAUT* Unified theory of acceptance and use of technology

^**a**^The opinions of the breast centre clinical leads were expressed as scores from 0 to 100

^**b**^Equality-of-means test

As regards effort expectancy as well as facilitating conditions, the scores were all lower and non-significantly different between the two groups of responses.

The clinical leads of breast centres collaborating with the screening programme expressed a greater agreement with the two statements concerning satisfaction and motivation, and agreed more often than their colleagues about the importance of integration of screening programmes into breast centres.

### Clustering of breast centres having a relation with the screening programme

Among the 62 valid questionnaires completed by professionals reporting some type of integration with screening programmes, six clusters of breast centres were identified based on the observed level of integration by dimension of integration. This choice was based on the agglomerative clustering and the distance between elements of each cluster.

Table 4 shows that the clusters were broadly distributed from ‘scarce’ to ‘high’ integration. The largest clusters were at the opposite extremities of the range. Twenty-five out of 62 centres (40%) were classified as having a poor to scarce level of integration.

**Table 4:** Type of integration in the six clusters and number of breast centres in each cluster

Cluster	Number of centres (%)	Type of integration
Fully integrated	12 (19)	High integration in all the three dimensions (structural, functional and process)
Highly integrated	13 (20)	High integration in structural and process integration, moderate functional integration
Moderately integrated	8 (12)	Moderate integration in all the three dimensions
Mildly integrated	6 (9)	Moderate structural integration, mild process and functional integration
Poorly integrated	19 (30)	Mild structural and process integration, moderate functional integration
Scarcely integrated	6 (9)	Scarce integration in all the three dimensions

Table 5 shows the results of cluster analysis. The six clusters reported significantly different average responses on selected items. Specifically, with respect to breast centre characteristics, those centres classified as highly integrated with a screening programme reported a higher number of breast radiologists (mean, 7.2 vs. 5.1,* P* < 0.04), number of radiology technicians or radiographers (mean 9.25 vs. 6.63,* P* < 0.08), and a greater mammogram reading volume (mean 35,330 vs. 21,268, *P* < 0.03).

**Table 5:** Cluster analysis of the observed correlates and effects of different levels of integration of screening programmes into breast centres according to clinical leads (*n* = 62)

	**Type of item**	**Mean (range)**	**SD**	**Equality-of-means test**		**Cluster(s) with a significantly different mean**			
				**F**	***P* value**	**Cluster**^**a**^	**Cluster** **mean**	**Mean of the other clusters**	***P* value**
Breast centre characteristics									
New breast cancer cases treated in the last year	Number	418.3 (345.1–656.5)	339.1	1.11	0.37	Medium	656.5	374.8	0.021
Dedicated breast radiologists	Number	5.5 (4.5–7.2)	3.2	1.19	0.33	High	7.2	5.1	0.039
Dedicated radiographers	Number	7.13 (5.15–9.25)	4.67	2.07	0.082	High	9.2	6.6	0.079
Mammogram reading volume in the last year	Number	24,595 (16,592–35,330)	21,076	1.31	0.27	High	35,330	21,268	0.031
Availability of a data manager	No/yes	0.78 (0.50–1.00)	0.42	1.98	0.096	Medium	1.00	0.72	0.088
Availability of a clinical database for quality assurance and research	No/yes	0.89 (0.50–1.00)	0.32	2.95	0.019	Medium–low	0.50	0.92	0.002
Performance expectancy									
The integration makes me more confident of the clinical quality of patient care	Score 1–100	93.4 (86.8–99.2)	14.20	1.49	0.21	Low	87.8	95.9	0.038
The integration makes me more confident of patient convenience (service timeliness, etc.)	Score 1–100	95.7 (89.0–100.0)	12.55	1.41	0.24	Low	90.9	97.8	0.046
The integration eases my job	Score 1–100	83.0 (43.8–96.2)	25.58	4.37	0.002	None	43.8	86.4	0.000
						High	96.2	79.8	0.046
The integration offers better opportunities for my professional growth	Score 1–100	73.7 (47.4–89.7)	29.40	2.22	0.065	None	47.4	76.0	0.036
						High	89.7	69.9	0.035
Effort expectancy									
It is easy to acquire the management skills needed for the integration	Score 1–100	55.3 (45.1–71.0)	27.56	1.78	0.13	Low	45.1	59.8	0.051
						High	71.0	51.5	0.027
Managing the integration does not cost me extra working time	Score 1–100	42.7 (32.6–62.6)	30.42	1.02	0.41	NC			
Social influence									
Do your colleagues think that the integration is important?	Score 1–100	85.7 (73.0–94.2)	20.57	1.09	0.37	NC			
Does local health authority think that the integration is important?	Score 1–100	76.2 (63.7–92.0)	27.93	1.34	0.26	Low	63.7	81.7	0.018
Facilitating conditions									
Has local health authority made available to you the resources needed for the integration?	Score 1–100	42.5 (23.2–62.2)	34.46	2.59	0.036	Low	23.2	51.0	0.003
Has local health authority enabled you to acquire the management skills needed?	Score 1–100	51.5 (36.7–67.3)	34.43	1.49	0.21	Low	36.7	58.0	0.023
						High	67.3	47.7	0.077
Has your local health authority developed an official protocol for the management of breast cancer?	Score 1–100	71.3 (48.4–90.1)	33.48	2.84	0.023	Low	58.3	77.1	0.040
						High	90.1	66.8	0.029
Propensity to use									
Are you inclined to handling the integration personally?	Score 1–100	92.6 (84.9 -100.0)	16.99	2.00	0.093	Low	85.5	95.8	0.027
Are you inclined to keep on handle the integration with conviction?	Score 1–100	92.6 (84.8–97.7)	16.52	1.17	0.33	NC			
Satisfaction and motivation									
The integration makes my working environment more stimulating	Score 1–100	85.1 (61.6–92.4)	20.03	2.59	0.036	None	61.6	87.2	0.005
The integration makes my working environment more satisfactory	Score 1–100	84.1 (66.2–93.8)	20.09	2.28	0.059	None	66.2	85.7	0.036
						High	93.8	81.8	0.065

*NC* No clusters (with a significantly different mean)

^a^None: scarcely integrated, low: poorly integrated, medium–low: mildly integrated, medium: moderately integrated, high: fully integrated. Only clusters showing significantly different mean values compared to the others were eligible for this analysis. This explains the absence of the medium–high level cluster. See Table 4 for details of the type of integration corresponding to each level

With respect to performance expectancy, the same centres reported a greater agreement that integration eases the job and offers better opportunities for professional growth. In parallel, centres with low or no integration disagreed more often with the statements that integration makes professionals more confident both of the clinical quality of patient care and of patient convenience, and that it offers better professional opportunities.

Regarding effort expectancy, the centres classified as highly integrated expressed a greater agreement that the management skills needed for the integration are easy to acquire, which was paralleled by an opposite finding among low-integration centres.

With respect to social influence, low-integration centres reported a significantly poorer agreement that peers think that the integration is important.

Unequivocal findings were obtained as to facilitating conditions, focusing on the role of local health authority. Highly integrated centres responded more often affirmatively to the question of whether the organisation did enable the clinical lead to acquire the management skills needed and did develop an official protocol for breast cancer care. The opposite occurred among low-integration centres.

As a final remark, the section of the questionnaire concerning ‘satisfaction and motivation’ showed that poorly integrated centres agreed less often with the statements that the integration makes the working environment more stimulating and more satisfactory. Regarding the latter, the clinical leads of highly integrated centres had an opposite opinion.

Incidentally, the cluster with the lowest level of integration assigned the lowest score to the question of whether the organisation made available the resources that are needed for the integration.

## Discussion

### Main findings

The above results may be summarised as follows. On the one hand, the proportion of breast centres reporting that they collaborate with the local screening programme was higher than expected, and was associated with the annual number of treated breast cancers and the BCCert certification.

On the other hand, however, the quality of integration varied greatly and was medium to low in most instances. Overall, the survey confirmed the positive effects of integrating screening programmes into breast units but, more important, the results brought to light some specific difficulties that must be faced.

### Main comments

Performance expectancy may be viewed as the degree to which the respondents believe that the integration has improved (or will improve) breast services, that is, its perceived usefulness. We evaluated four indicators and found that they were all significantly higher in those breast centres integrating a screening programme, which was also the case for the indicators of satisfaction and motivation. The statement that integration makes more confident both of the clinical quality and the convenience of patients and eases the job was largely agreed upon, indicating that there are beneficial effects for the staff of breast centres as well as the attending women.

The findings were different for effort expectancy indicators, which measure the degree to which the responding breast centre clinical leads believe that the integration is easy to implement. The scores were lower in both groups of responses. This means that feasibility concerns may discourage those who have never undertaken the integration and are shared by those clinical leads who did it. The many problems affecting the creation of breast centres in all European countries (in particular, lack of national laws and plans, lack of resources, incomplete accreditation by independent agencies, absence of mandatory requirements, etc.) have been described [6].

This consideration brings us to another predictable finding. Those survey participants who had experienced the integration of a screening programme reported that no particular facilitating conditions were promoted for the integration. Compared with respondents reporting no integration, they did not receive more institutional support with respect to resources and managerial training. Unexpectedly, the availability of an official protocol for the management of breast cancer, which is expected to facilitate the unification of breast services, had not any such effect. Overall, these findings would suggest that a successful integration depends greatly on the personal commitment of the clinical lead rather than reflecting institutional strategies and policies. To some extent, this confirms the view publicly expressed by the GISMa [10] that an insufficient integration of screening programmes into breast centres might often depend on an insufficient commitment of clinicians involved in breast care to work with a fully multidisciplinary approach, with the active participation of screening staff.

Cluster analysis was performed in order to obtain a more detailed representation of the many and multifaceted differences associated with different levels of integration of screening programmes. The results confirmed the marked heterogeneity of Italian breast centres. A higher number of breast radiologists and radiographers and a greater mammogram reading volume were expected characteristics of those breast centres classified as highly integrated with a screening programme. Also, this cluster reported a significantly higher agreement that integration eases the job, offers better opportunities for professional growth and makes the working environment more satisfactory. Noteworthy, the availability of an official protocol for the management of breast cancer and the local health authority’s support, which did not discriminate significantly between centres integrating and non-integrating a screening programme (see above), were two correlates of this cluster alone. These are key observations, because they indicate two essential prerequisites for an optimal integration.

The clusters with the lowest level of integration or no integration expressed a lower level of agreement with most statements, with a combination of poor expectancies, poor social support, poor facilitating conditions, and poor propensity. The –by far– lowest score was assigned to the question of whether local health authority has made available the resources that are needed. This is another finding worthy of consideration.

### Methodological considerations

There are some major methodological issues in this study that need to be addressed. The first regards the representativeness of participating centres. Since an official national list of breast centres was not yet available, we targeted for the survey the pool of centres associated with Senonetwork, which we consider to represent an acceptable approximation. The response rate was 65%, a figure sufficient to ensure that the validity of results is not threatened by a substantial nonresponse bias. Two-thirds of responding centres were situated in northern Italy, but this reflects the higher density of breast centres and the two-fold greater prevalence of active and efficient local screening programmes in the north of the country, with > 85% of women aged 50–69 years being regularly invited to mammography versus approximately 40% in southern Italy [27, 28]. The number of new breast cancer cases per year was above 150 (minimum acceptable standard according to the EUSOMA) in all centres but one. However, this confirms an increasing trend recently reported [28] rather than suggesting a selection bias. With respect to the proportion of regionally appointed centres, the observed figure was 74% and will most likely increase further.

The second methodological issue to consider is that this study is a cross-sectional survey. Consequently, we cannot exclude the biases related to this kind of design [29, 30]. To reduce this possibility, however, the questionnaire adopted different scales and the results were discussed with experts who are aware of the characteristics of the centres.

Third, a reason for caution in interpreting the results is that the operational difficulties caused by the COVID-19 crisis has led to a delay in the roll-out of the survey after pilot and in the discussion of the findings before publication. For the same reason, however, it is highly unlikely that the observed problems have been overcome –or at least partially addressed and remedied– since the survey was done. Many countries, including Italy, have suspended or limited cancer screening services as part of their response to the pandemic [31].

### Policy implications

As regards policy implications, it is important to note that the deficiency of facilitating conditions (resources, acquisition of the management skills needed, and development of an official protocol for the management of breast cancer) is modifiable. In particular, screening professionals’ societies may have a role as initiators of the integration, and this role can be boosted by sharing the screening principles with the clinical counterpart and by promoting research and education initiatives in a multidisciplinary environment. Other supporting actions can be included in health laws at the national and regional level, such as raising the minimum activity volume threshold for breast centres, making an official integration protocol available, and providing support, resources and managerial education.

## Conclusions

The results of the survey provided insights on the importance of the integration of screening programmes into breast centres focusing on the perspectives of professional career and motivation. This is a pivotal element in the process of facilitating the organisational innovation. Although requiring additional effort, integration seems to be a powerful lever to promote professional interests. The deficiency of facilitating conditions is modifiable. Further research will have to update the baseline described here and give due attention to elucidating the relationship between the degree and dimension of integration and the whole breast cancer care pathway.

## Data Availability

The datasets generated and analyzed during the current study are not publicly available due to Ethics Committee restrictions but are available (in Italian) from the corresponding author upon reasonable request.

## Abbreviations

EUSOMA: European Society of Breast Cancer Specialists
GISMa: *Gruppo Italiano Screening Mammografico* (Italian Group for Mammography Screening)
UTAUT: Unified theory of acceptance and use of technology
